# Comparison of three imaging modalities used to evaluate bone healing after tibial tuberosity advancement in cranial cruciate ligament-deficient dogs and comparison of the effect of a gelatinous matrix and a demineralized bone matrix mix on bone healing – a pilot study

**DOI:** 10.1186/s12917-018-1490-4

**Published:** 2018-05-22

**Authors:** Marije Risselada, Matthew D. Winter, Daniel D. Lewis, Emily Griffith, Antonio Pozzi

**Affiliations:** 1 Department of Small Animal Clinical Sciences, College of Veterinary Medicine, University of Florida, Gainesville, FL 32610-0126 USA; 2 Present address: Department of Veterinary Clinical Sciences, College of Veterinary Medicine, Purdue University, Lynn Hall, 625 Harrison Street, West Lafayette, IN 47907 USA; 3 Department of Statistics, College of Agriculture and Life Sciences, North Carolina State University, Raleigh, NC USA; 4 Present address: Department for Small Animals, Vetsuisse University of Zurich, Winterthurerstrasse 258c, 8057 Zurich, Switzerland

**Keywords:** Osteotomy, Bone healing, Ultrasound, CT, TTA

## Abstract

**Background:**

Bone healing and assessment of the state of bone bridging is an important part of clinical orthopedics, whether for fracture healing or for follow up of osteotomy procedures. Tibial tuberosity advancement (TTA) is designed to restore stability in cruciate deficient stifle joints by advancing the tuberosity while creating an osteotomy gap. The current study aims to: 1) compare three different imaging modalities to assess bone healing: ultrasound, radiographs and computed tomography (CT) and, to 2) compare the effect of a gelatinous matrix (GM) versus a demineralized bone matrix mix (DBM mix) on bone healing and bridging of this osteotomy gap in 10 otherwise healthy client-owned dogs with cranial cruciate ligament insufficiency. Osseous union of the osteotomy gap was evaluated with ultrasound, radiographs and CT at one, two, and 3 months postoperatively. Dogs were randomly selected to receive GM or DBM mix to fill the osteotomy gap created during the TTA procedure. Bone healing was assessed subjectively on all modalities as well as scored on radiographs and measured using Hounsfield units (HUs) on CT. Time to heal based on ultrasound, radiographs and CT were statistically compared between groups with significance set at *p* < 0.05.

**Results:**

All osteotomy gaps were bridged with bone within 3 months for all modalities. Bridging bone was diagnosed in 5.6 weeks, 10.4 weeks and 9.6 weeks based on ultrasound, radiographs, and CT, respectively, in dogs treated with DBM mix. In dogs treated with GM osseous union was diagnosed in a mean of 4.0 weeks, 9.6 weeks and 7.2 weeks based on ultrasound, radiographs and CT. Ultrasound diagnosed osseous union significantly faster than both CT and radiographs (*p* < 0.001). The dimensions of the newly formed bone differed between treatment groups with the central portion of the bone only providing a small bridge in GM cases. Although bridging of the osteotomy gap occurred earlier in the group that received GM, no significant statistical difference was found between the two groups.

**Conclusions:**

Radiographs overestimate the time needed for osseous union of the osteotomy gap. All osteotomy sites healed radiographically within 3 months.

## Background

The tibial tuberosity advancement (TTA) procedure aims to restore stability in the cranial cruciate ligament deficient stifle by advancing the patellar ligament cranially. The resulting decrease in patellar tendon angle causes a caudal shift in the femorotibial shear force, which has been shown to eliminate the cranial subluxation of the tibia in cadaveric ex vivo models [1, 2]. Clinically the TTA procedure has gained acceptance as a surgical option to address stifle instability in cranial cruciate ligament-deficient stifles, and the efficacy of this procedure has been documented in several studies [3–8]. During the TTA (Kyon, Technoparkstrasse 1, Zurich, Switzerland) procedure, a cage ranging in size from three to 15 mm is inserted to maintain the tibial tuberosity in the advanced position. This advancement creates a wedge shaped osteotomy gap. Recommendations for managing the gap caused by cranial advancement of the tibial tuberosity include filling the osseous defect with autogenous cancellous bone [3–5, 9] or allogenic bone graft substitutes such as demineralized bone matrix mixed with allogenic corticocancellous bone (DBM mix: Freeze dried Cancellous Demineralized Standard mix (1cm^3^) Veterinary Transplant Services, Inc. (Kent WA, USA)) [3]. Use of a cancellous bovine bone graft material (bovine xenoimplant) has been recently described [9]. Other authors have suggested that filling the gap with autologous cancellous bone or suitable graft substitute may not be necessary [8, 10, 11]. At the time of this study gelatinous matrix (GM)(TRMatrix™, IMEX™ Veterinary Inc., Longview, TX) was a commercially available bone graft substitute that resembles tertiary embryonic connective tissue. This material was purported to have osteopromotive properties [12, 13] but the clinical efficacy of this GM has not been previously described in a case series or prospective study. It is currently not available for purchase, and was discontinued for the veterinary market due to limited volume [14].

Traditionally, radiographs have been used to assess bone formation following fracture repair or osteotomy procedures [1, 3–6, 8–11]. Several definitions of radiographic union have been reported, generally using cortical bridging as an indication for a healed osteotomy [8–11]. Other imaging modalities available to assess bone healing include ultrasonography (US) [15–17] and computed tomography (CT) [18, 19]. Ultrasonography has been used to detect (occult) fractures, evaluate bone production in limb-lengthening procedures and to evaluate fracture healing [15–17, 20–26]. Based on several studies, US detects tissue bridging earlier than radiography [15–17, 20–26]. Ultrasonographic union has been defined as a complete hyperoechic bridge with acoustic shadowing and validated using histology [15–17]. Although CT is rarely used to diagnose fracture healing with only two prospective studies published [18], it has been extensively used to follow up bone osteogenesis [27–30]. Furthermore, it can quantify bone healing by measuring Hounsfield units (HUs) [19, 27], which could make it an excellent method to evaluate an osteotomy union objectively. The current study was designed to compare utility of these three different imaging modalities for assessing new bone formation in a standardized clinical osteotomy gap model. Our first objective was to compare the time to osteotomy union using US, radiography and CT based on previously reported definitions of union [8–11, 15–18]. As a second objective, we wanted to compare the osseous response induced by placing two different bone graft subsitutes in the TTA osteotomy site. Our hypotheses were that: 1) all osteotomy gaps would be bridged by bone based on all three imaging modalities by the end of the study (3 months), 2) US would substantiate osteotomy union earlier than the other two imaging modalities, and that 3) GM would promote faster healing than DBM mix.

## Methods

### Animals

Ten healthy middle aged, medium or large breed client owned dogs with naturally occurring unilateral cranial cruciate ligament insufficiency were enrolled in the study. Five of the enrolled dogs were randomly assigned to the GM treatment group (TRMatrix™, IMEX™ Veterinary Inc., Longview, TX) and the other 5 dogs were assigned to the DBM mix treatment group (Freeze dried Cancellous Demineralized Standard mix (1cm^3^); Freeze-Dried Osteo-Allograft (1cm^3^) Veterinary Transplant Services, Inc. (Kent WA, USA). Approval for the study was granted by the University of Florida Institutional Animal Care and Use Committee (#200801191) according to the guidelines by the Animal welfare act and US Government principles for the utilization and care of vertebrate animals. Written informed owner consent was obtained according to the guidelines of and with approval of the University of Florida, College of Veterinary Medicine Clinical Research Review Committee.

### Surgery

The required tuberosity advancement and cage size were determined prior to surgery according to overlay templates (Kyon, Technoparkstrasse 1, Zurich, Switzerland) [3]. Prior to performing the osteotomy, an arthrotomy or arthroscopy was performed to assess the integrity of the cruciate ligaments, menisci and articular cartilage. Remaining fibers of the cranial cruciate ligament were debrided. Cranial cruciate ligaments with only minor fiber pathology resulting in that stifle having minimal cranial tibial thrust and drawer were classified as partial ruptures and the cranial cruciate ligament was not debrided. Menisci with gross parenchymal pathology were treated appropriately by performing a caudal pole hemimeniscectomy or partial meniscectomy. The decision to perform a meniscal release was based on surgeon preference and was documented in the medical record.

The osteotomy was performed using an oscillating saw, with continuous saline irrigation. An appropriately sized fork-plate combination was used and an appropriately sized cage was placed in the osteotomy gap [3, 4]. The osteotomy gap was lavaged copiously with sterile saline before placing either GM (1 vial of 1 cm^3^) or DBM mix (1 cm^3^) in the defect. One vial of GM was used to fill the defect, making sure not to place any product outside the osteotomy gap. One cc of DBM mix was used to fill the osteotomy gap. Closure of the surgery site was performed routinely. All surgeries were performed by the same surgeon (MR). Appropriate advancement of the tuberosity and position of the implants was assessed on orthogonal view radiographs obtained under anesthesia prior to recovering the dog.

All dogs had a modified Robert Jones bandage placed on the limb for 24 h following surgery. This bandage was removed the day after surgery. The bandage was not reapplied in some dogs or was reapplied and subsequently removed within 5 days after surgery. Owners were instructed to perform passive range of motion exercises for the first four to 6 weeks after surgery. All dogs received opioid analgesia: either methadone hydrochloride (Methadone, 10 mg/ml, Mylan Institutional LLC, Rockford, IL) 0.1–0.2 mg/kg IV q4-6 h or hydromorphone hydrochloride (Dilaudid, 2 mg/ml, West-Ward Pharmaceuticals, Eatontown, NJ) 0.05–0.1 mg/kg IV q6-8 h for the first 24 h after surgery, followed by tramadol hydrochloride (Ultram, Janssen Pharmaceuticals, Titusville, NJ) 2.2 mg/kg PO q8-12 h for five to 10 days. All dogs received additional non-steroidal anti-inflammatory drug (NSAID) analgesia at an appropriate dose unless contra-indicated as judged on a chemistry panel. Drugs administered were: deracoxib (Deramaxx© Novartis Animal Health, Greensboro, NC) or carprofen (Rimadyl® Pfizer Animal Health, Lincoln, NE) daily for the first 4 weeks and on an as needed basis thereafter, as judged on a day to day basis by the owner.

All dogs were confined to a crate or cage when unattended and restricted to leash walks when outdoors for the first 3 months following surgery. Owners were instructed to return their dogs to be re-evaluated and imaged at four, eight and 12 weeks following surgery.

### Imaging modalities

#### Radiographs

Orthogonal view radiographs (CR) were obtained immediately postoperatively and at the four, eight, and 12 week re-evaluations using a computed radiography system (Kodak/Carestream, Directview, Carestream Health, Inc., Rochester, NY). All lateral projection radiographs were obtained with the stifle positioned in an approximate flexion angle of 135°.

Radiographic evidence of healing included progressive increase in mineral opacity in the osteotomy gap, with bridging of the osteotomy margins and confluence of the osteotomy segments. The lateral view radiographs were also scored on a scale from ‘0–4’ using a previously published semi-quantitative scoring system [4] (Hoffman scores): ‘0’ = no bone healing in any area; ‘1’ = early bone healing, no bridging between the tibial tuberosity and tibial shaft; ‘2’ = bridging bone at one site; ‘3’ = bridging bone at two sites; ‘4’ = bridging bone at three sites (proximal to the cage, between cage and plate, distal to the plate) [4]. These scores were used for temporal comparisons in individual dogs and between treatment groups. A 12-step metal stepwedge was placed in the primary beam and adjacent to the limb when obtaining the lateral view radiographs as an objective evaluation method to assess the radiographic opacity of the tissue in the osteotomy gap. The opacity of the tissue in the osteotomy gap at the aforementioned three sites (proximal to the cage, between cage and plate, distal to the plate) was compared to the steps. The tissue was assigned the step number of which the radiographic appearance most closely matched the radiographic appearance of the evaluated tissue in the osteotomy site by visual overlay. Visualization of the cortical chips in the DBM mix treatment group in the osteotomy gap was noted as visible or non-visible.

#### Computed tomography

CT examinations were performed immediately postoperatively and at the four, eight, and 12 week re-evaluations using an 8 slice multi-detector row computed tomography unit (Toshiba Acquilion 8, Toshiba America Medical Systems, Tustin, CA). Dogs were positioned in dorsal recumbency with the hind limbs extended to position both stifles at approximate a 135° angle. Contiguous 2 mm axial slices of both hind limbs were obtained from the mid femur to the distal tibia. The volume data was stored on disks and the rendered studies were stored on a picture archive and communication system (PACS) (AMICAS PACS™ Merge® Healthcare, Morrisville, NC). Measurements were performed using software proprietary to the PACS. Healing of the osteotomy on CT studies was subjectively assessed based upon volume and progression of mineral attenuating tissue within the osteotomy gap, bridging of the osteotomy margins and continuity of the osteotomy segments. Attenuation within the osteotomy gap was objectively assessed by measuring the CT numbers within a best fit oval region of interest (ROI) that only included the tissue within the osteotomy gap, drawn on all axial slices, starting immediately distal to the cage and continuing to the distal most portion of the osteotomy gap. The mean of these measurements was calculated for every CT examination and these values were used for temporal comparisons in individual dogs and between treatment groups. The minimal width of the osseous bridge in the osteotomy gap distal to the cage was measured using a digital caliper, with the points placed at the medial and lateral margins of the bone-soft tissue interface. To account for size differences between dogs, a ratio of the following measurements was used: 1) the width of the osteotomy line at the narrowest point, 2) width at the cut edge of the tibial tuberosity on the same axial slice, and 3) the width at the cut edge of the tibial diaphysis was on the same axial slice. The ratio was calculated as follows:

$$ ratio=\frac{width\kern0.17em of\; new\; bone\kern0.17em formation}{\left( width\kern0.17em of tibial\kern0.17em tuberosity+ width\kern0.17em of\kern0.17em tibial\kern0.17em diapysis\right)/2} $$

#### Ultrasonography

B-mode ultrasonography was performed at four, eight and 12 week re-evaluations through a medial and lateral window centered over the osteotomy gap using a 5–17 MHz linear broadband transducer (Philips iU22, Philips Healthcare, Andover, MA). No ultrasound was performed immediately after surgery due to expected interference with air in the surgery site. Ultrasonographic criteria that were used to assess healing were taken from prior work [15–17, 21]. The following criteria were used: 1) the tissue in the osteotomy gap had a hyperechoic interface with presence of distal acoustic shadowing indicating mineralization of the tissue and consistent with new bone formation, 2) the hyperechoic tissue in the osteotomy gap was continuous with the tibial tuberosity cranially and the tibial shaft caudally indicating bridging of the osteotomy gap. Ultrasonographic studies were scored subjectively as sonographically healed or non-healed.

#### Comparisons

The two treatment groups were assessed independently for time until bony bridging for each individual modality. A diagnosis of a healed osteotomy gap by bridging bone for each individual modality was determined as defined by the parameters outlined above. This time point was used for comparison between the modalities. For this comparison, the two treatment groups were combined into one patient group.

#### Statistical analysis

The mean number of weeks until bony bridging was identified for each of the different imaging modalities (CR, CT and US). These values were used to compare the subjective assessment of osteotomy union between imaging modalities. The specific criteria outlined above were used to define healing. Both surgical treatment groups were combined in this assessment to provide a more robust group. The comparison was performed using the Cochran-Mantel-Haenzel tests for a difference in row mean scores. Within each imaging group the effect of imaging modalities and treatment (GM v. DBM mix) were tested using the same methodology. The radiographic scores based on the Hoffman grading system were also analyzed using Cochran-Mantel-Haenzel tests for a difference in row mean scores between treatments (GM v. DBM mix) at each time point. The HUs on the CT images were analyzed using a repeated-measures linear model to test for treatment and time effect.

A Kruskal-Wallace nonparametric test was used to compare the ratio of the width of new bone growth to the tibial width between the two treatments. Significance was defined as values of *p* < 0.05. All statistical analyses were performed by use of computer software (SAS, version 9.3; SAS Institute Inc., Cary, NC).

## Results

### Animals & surgery

The mean age of the dogs was 71 ± 15.8 months and the bodyweight 34.4 ± 8.2 kg. No significant differences were found between the two treatment groups for age (*p* = 0.18), bodyweight (*p* = 0.18), or cage size (*p* = 0.18). All patient parameters are summarized in Table 1. Seven dogs received daily NSAIDs until the first recheck at 4 weeks, two dogs did not receive NSAIDs (one in each treatment group) and the remaining dog received daily NSAIDs for 8 weeks (DBM group).

**Table 1:** Breed, Age, Body weight (BW), MM (medial meniscal treatment) of dogs treated with DBM (D1–5) and dogs treated with GM (G1–5): CHM (caudal hemimeniscectomy), MR (meniscal release); cage size (in mm) and additive: DBM mix (demineralized bone matrix mix), or GM (gelatinous matrix) for the two treatment groups

	Breed	Age (months)	BW (kg)	CrCL	MM	Cage (mm)	Additive
D1	Mix	96	33.2	Partial	CHM	12	DBM
D2	Labr. R.	59	40	Complete	MR	9	DBM
D3	GSD	64	45	Complete	CHM	12	DBM
D4	Labr. R.	91	39	Complete	CHM	12	DBM
D5	Mix	69	27	Partial	None	12	DBM
Mean		75.8 ± 16.6	36.8 ± 6.9				
G1	Mix	48	30	Complete	CHM	9	GM
G2	Labr. R.	87	48	Complete	MR	12	GM
G3	Sharpei	56	23	Complete	CHM	9	GM
G4	Labr. R.	72	30	Complete	CHM	9	GM
G5	Mix	68	28.6	Partial	None	12	GM
Mean		66.2 ± 15	31.9 ± 9.4				
Total		71 ± 15.8	34.4 ± 8.2				

### Imaging

#### Radiographic assessment

**Fig. 1:**
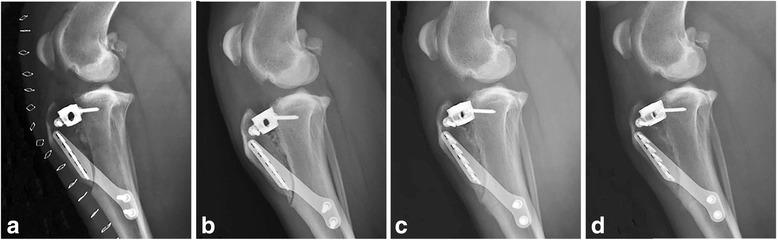
Four mediolateral projections of the left stifle of a dog in the DBM mix group made with the stifle in 135° of flexion. (**a**) immediately postoperatively, (**b**) 1 month postoperatively, (**c**) 2 months postoperatively; (**d**) 3 months postoperatively. Note the progressive, heterogeneous increase in mineral opacity within the osteotomy gap over time, with progressive sclerosis of the osteotomy margins consistent with healing. On images (**b**-**d**), the patellar ligament is thickened. The increased soft tissue within the stifle joint and the moderate osteophyte production remain relatively static over time

**Fig. 2:**
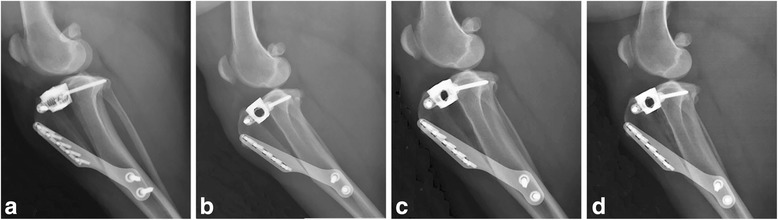
Four mediolateral projections of the right stifle of a dog in the GM group made with the stifle in 135° of flexion. (**a**) immediately postoperatively, (**b**) 1 month postoperatively, (**c**) 2 months postoperatively; (**d**) 3 months postoperatively. Note the less pronounced, but still progressive increase in mineral opacity within the osteotomy gap over time. On images (**b**-**d**), the patellar ligament is thickened. The increased soft tissue within the stifle joint and the minimal osteophyte production remain static over time

Air was seen in the osteotomy gap in all immediate postoperative radiographs. Cortical bone chips were easily identified immediately postoperatively in the DBM mix treatment group (Fig. 1). The tissue in the osteotomy gap in the GM treatment group had a radiographic appearance similar to soft tissue (Fig. 2). No complications associated with the implants were seen at any of the recheck radiographs. Bone chips could still be identified at 3 months postoperatively in two of five DBM mix dogs (Fig. 1). The bridging bone at final recheck had a smooth trabecular pattern in all GM cases. All of the TTAs had achieved complete radiographic union at 3 months. Healing times are summarized in Table 2.

**Table 2:** Outcomes of dogs treated with DBM mix (D1–5) and dogs treated with GM (G1–5). Healing diagnoses are listed subjectively as ‘healed’ or ‘not-healed’ and expressed as weeks postoperatively (i.e. the recheck in weeks postoperatively (PO) where the diagnosis was ‘healed’. Individual cortical chips visible, qualitatively scored as yes or no, and expressed in months postoperatively. US: ultrasonography; CT: computed tomography; CR: computed radiography. The total healing time is expressed as mean [median]. There is a large difference between methods, but not between treatments by methods (for CR, *p* = 0.655; for CT, *p* = 0.578; for us, *p* = 1.000). * US healing times significantly different from both CT and CR (*p* <0.05)

	Complete Healing (weeks PO)			Visibility of bone chips (weeks PO)	
	US	CT	CR	CT	CR
D1	8	12	12	12	12
D2	8	8	12	8	4
D3	4	8	8	4	4
D4	4	8	8	8	8
D5	4	12	12	12	12
DBM mix	5.6	9.6	10.4		
G1	4	8	12	n/a	n/a
G2	4	8	12	n/a	n/a
G3	4	8	8	n/a	n/a
G4	4	4	4	n/a	n/a
G5	4	8	12	n/a	n/a
GM	4	7.2	9.6		
Total	4.8^*^	8.4	10		

The median Hoffman [4] score was ‘0’ immediately after surgery and progressed to a median score of ‘3’ at 3 months in both groups (Table 3). The median stepwedge number associated with the tissue in the osteotomy gap was scored as a ‘1’ for all four time points for both treatment groups (Table 4).

**Table 3:** Presented are the healing outcomes of dogs treated with DBM (D1–5) and dogs treated with GM (G1–5), based on scores (0–4) on lateral radiographs, as previously described by Hoffman et al. [0 = no bone healing in any area; 1 = early bone healing, no bridging between the tibial tuberosity and tibial diaphysis; 2 = bridging bone at one site; 3 = bridging bone at two sites; 4 = bridging bone at 3 sites (proximal to the cage, between cage and plate, distal to the plate)] [4]. No significant differences were found between treatment groups at any time point or over time

	Scores			
	0w PO	4w PO	8w PO	12w PO
D1 (1)	0	1	2	3
D2 (5)	0	2	3	3
D3 (7)	0	1	3	4
D4 (9)	0	3	3	3
D5 (11)	0	1	–	3
Median	0	1	3	3
G1 (2)	0	3	3	3
G2 (4)	0	2	3	3
G3 (6)	0	1	3	3
G4 (8)	0	3	–	3
G5 (10)	0	1	3	3
Median	0	2	3	3

**Table 4:** Outcomes of dogs treated with DBM mix (D1–5) and dogs treated with GM (G1–5) based on stepwedge numbers. The stepwedge numbers ranged from 1 to 12, with one being the least dense and 12 the highest density possible. Comparisons were made at three sites: proximal to the cage, between cage and plate, distal to the plate, and the mean is reported. No significant differences were found between time points or treatment groups

	Stepwedge numbers			
	0w PO	4w PO	8w PO	12w PO
D1 (1)	1	1	1	1.67
D2 (5)	2	1	1	2
D3 (7)	1	1	1	1
D4 (9)	1	1	1	1
D5 (11)	1	1	–	1
G1 (2)	1	1	2	2
G2 (4)	1	1	1	2
G3 (6)	1	2	1	1
G4 (8)	1	1	–	1
G5 (10)	1	1	1	1

#### Computed tomographic assessment

**Fig. 3:**
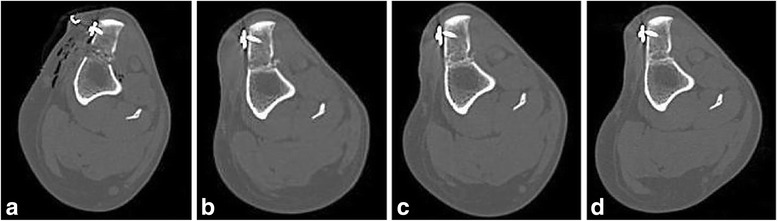
Four axial computed tomographic (CT) images of the left stifle of the same dog in Fig. 1 (DBM mix group) made at the level of the first prong of the fork. (**a**) immediately postoperatively, (**b**) 1 month postoperatively, (**c**) 2 months postoperatively; (**d**) 3 months postoperatively. Note the progressive increase in heterogeneous mineral attenuation with near complete filling of the osteotomy gap. On image (**d**), there is smooth, contiguous bone at the medial aspect of the osteotomy

**Fig. 4:**
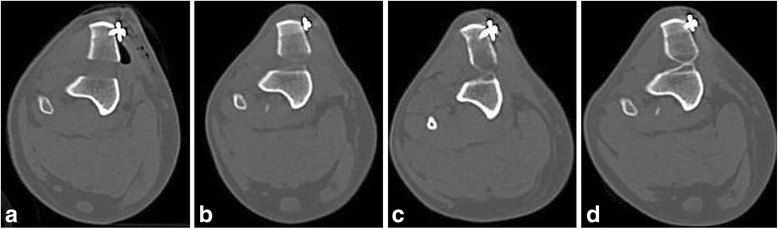
Four axial computed tomographic images of the right stifle of the same dog in Fig. 2 (GM) made at the level of the first prong of the fork. (**a**) immediately postoperatively, (**b**) 1 m postoperatively, (**c**) 2 months postoperatively; (**d**) 3 months postoperatively. Note the slow progressive increase of smooth, more homogenous mineral attenuation that becomes more well-defined on Image (**d**), consistent with healing. This smooth bone production tapers toward the center of the osteotomy, forming an hourglass shape, and resulting in incomplete filling of the osteotomy gap

On the immediate postoperative studies, air could be seen dissecting along the fascial planes in all cases, and along the caudal cortex of the femur at the level of the fabellae in eight out of 10 dogs. Bone chips were easily identified in the DBM mix group in the immediate postoperative CT (Fig. 3). Transverse CT images showed progressive alterations of the bone graft substitutes in the osteotomy gap characterized by progressive increase in mineral attenuation in both groups. This increase was heterogenous and irregular in the DBM mix group. The mineralization in the GM group was smooth, and while bridging of the osteotomy gap was achieved, this process was (subjectively) less exuberant than the DBM mix group with a distinctive hourglass shape, resulting in less complete filling of the osteotomy gap (Fig. 4). Complete healing, or bony bridging on CT, of the osteotomy gap as defined by the volume and mineral attenuating nature of the tissue in the osteotomy gap, was diagnosed at 2 months postoperatively in three dogs and 3 months postoperatively in two dogs in the DBM mix treatment group. In the GM treatment group, complete bony bridging of the osteotomy gap on CT was diagnosed at 1 month postoperatively in one dog and at 2 months in the other four dogs (Table 2).

**Fig. 5:**
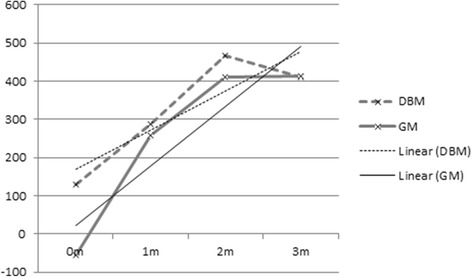
The Y axis shows the density in Hounsfield units, and the time postoperatively is plotted on the X-axis. A trendline (thinner line) is shown for both the DBM mix and the GM

The width of the bone at the thinnest point of the bridge was significantly smaller in the GM group (*p* = 0.03) The mean of the ratios for the GM treatment group was 0.48 (range 0.28–0.78) whereas the mean for the DBM mix treatment group was 0.79 (range 0.6–0.93) (Table 5). For the width ratios, there was not a statistically significant difference at the 0.05 level (*p* = 0.10). The grouped measurements for the HUs increased sharply between imaging immediate postoperatively and at 4 weeks, but leveled off thereafter (Fig. 5, Table 6). There were no differences between treatment groups after the initial measurement (*p* = 0.011 at time 1, *p* > 0.05 for all other weeks), but there was a statistically significant increase in HUs over time for all dogs (*p* = 0.047).

**Table 5:** Outcomes of dogs treated with DBM mix (D1–5) and dogs treated with GM (G1–5). Presented are the results based on Computed Tomographic imaging. ‘Width bridge’ is the width of the narrowest portion of the bony bridge in the osteotomy gap (in mm). ^1^Ratio of the narrowest bridge and the original cut. No significant differences were found between the two treatment groups (*p* = 0.1)

	Width bridge (mm)	Ratio bridge to cut^1^
D1	8.1	0.86
D2	11.2	0.93
D3	8.9	0.83
D4	4.0	0.60
D5	5.7	0.71
Mean	7.58	0.79
G1	5.6	0.75
G2	3.6	0.33
G3	3.3	0.24
G4	4.9	0.78
G5	2.6	0.28
Mean	4.0	0.48

**Table 6:** Healing outcomes of dogs treated with DBM mix (D1–5) and dogs treated with GM (G1–5), measured in Hounsfield units (HUs) on a PACS, using a best fit elliptical fit on the osteotomy gap on all available axial slices and presented as a mean of all measurements. The HUs increased significantly over time (*p* = 0.047)

	Bone density on CT (HUs)			
	0w PO	4w PO	8w PO	12w PO
D1 (1)	147.54	213.33	395.37	384.13
D2 (5)	134.11	313.01	637.65	666.44
D3 (7)	58.83	354.22	445.02	370.69
D4 (9)	43.31	366.95	386.45	508.67
D5 (11)	261.19	186.14	–	125.24
G1 (2)	− 106.04	457.39	696.20	741.30
G2 (4)	− 42.64	267.09	470.10	501.85
G3 (6)	31.84	193.54	433.48	484.71
G4 (8)	−71.76	315.95	–	305.48
G5 (10)	−85.10	55.74	42.28	38.67

#### Ultrasonographic assessment

**Fig. 6:**
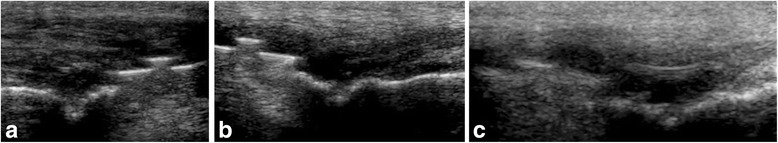
Three ultrasonographic images made of the osteotomy site of the same dog in Figs. 1 & 3. (**a**) 1 m postoperatively, (**b**) 2 months postoperatively; (**c**) 3 months postoperatively. Note the presence of strongly reflective, irregular interfaces within the osteotomy gap indicative of bone production, and compatible with healing of the osteotomy in all images

**Fig. 7:**
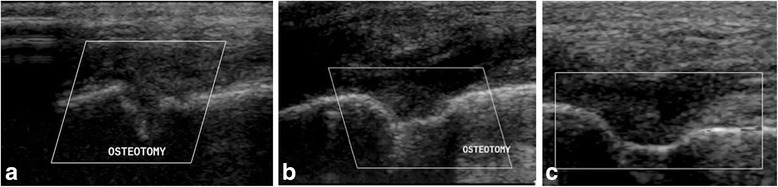
Three ultrasonographic images made of the osteotomy site of the same dog in Figs. 2 & 4. (**a**) 1 m postoperatively, (**b**) 2 months postoperatively; (**c**) 3 months postoperatively. Note the presence of strongly reflective, smooth, concave (hourglass) interfaces within the osteotomy gap indicative of bone production, and compatible with healing of the osteotomy in all images

The osteotomies were diagnosed as bridged with bone between one to 2 months postoperatively (Table 2). The bridging mineral present in osteotomy gaps of the GM treatment group had a smooth, but concave interface that was a feature of the hourglass shape noted on CT. The bridging mineral present in the osteotomy gaps of the DBM mix group was moderately irregular, without evidence of concavity, suggestive of the more complete filling noted on CT (Fig. 6). All five dogs in the GM treatment group were diagnosed as completely healed at their 4 week recheck (Fig. 7).

### Comparisons

#### Imaging modalities

Subjective assessment of time until osseous union differed significantly between the three evaluation methods (*p* = 0.001). The time until osseous union was diagnosed by CT and radiographs did not differ significantly (*p* = 0.28), but the time until ultrasonography diagnosed bone bridging with return of acoustic shadowing differed significantly from both an assessment of bone bridging on CT (*p* = 0.005) and on radiographs (*p* < 0.0001). When separated by imaging modality, the week that healing was first detected does not differ between the two treatment groups (radiographs, *p* = 0.655; CT, *p* = 0.578; ultrasonography, *p* = 1.00).

#### Comparison of treatment groups

No significant difference was found between treatment groups for time until a subjective assessment of radiographic healing was given (*p* = 0.655). There were no significant differences in Hoffman scores between the two treatment groups at any of the time points (1 month postoperatively (*p* = 0.501), and 2 months postoperatively (*p* = 0.76), and all but one dog had a score of ‘3’ at 3 months postoperatively), nor was a significant difference found in time until a score of ‘3’ was obtained (*p* = 0.259). No significant differences were found between time points for the stepwedge number assigned at any time point. No significant difference was found between treatment groups for time until a subjective assessment of healing was given on CT (*p* = 0.578). There was a statistically significant increase in HUs over time (from 0 to 3 months) for all dogs (*p* = 0.047) (Table 6).There were no differences between treatment groups for measured HUs in the osteotomy on CT after the initial measurement (*p* = 0.01 at time 1, *p* > 0.05 for all other weeks). Complete bridging with return of acoustic shadowing (ultrasonographic diagnosis of healing) was not significantly statistically different between treatment groups (*p* = 1.00)(Table 2).

## Discussion

The hypotheses tested during this study were that: 1) all osteotomy gaps would be bridged by bone based on all three imaging modalities by the end of the study (3 months), 2) US would substantiate osteotomy union earlier than the other two imaging modalities, and that 3) GM would promote faster healing than DBM mix. The results of this study showed that all of the osteotomy gaps in the dogs obtained osseous union without complications within 3 months, which validated our first hypothesis, that both treatment groups would progress to complete union within the study time frame. Obtaining histological confirmation of osseous union would have strengthened this study, but was not deemed appropriate given the study population (client owned animals), and potential for surgical morbidity. A significant difference was found between CT, radiographs and ultrasound with ultrasound being able to provide a diagnosis of healing earlier than radiographs or CT, confirming our second hypothesis. No significant differences were found for healing times between the two treatment groups, rejecting our third hypothesis. A power analysis revealed that least 62 dogs would be needed to reach significance, based on the most sensitive modality (CT).

We elected to use a 3 month postoperative follow-up period in this study for all three imaging modalities as previously published reports have shown that the majority of TTA osteotomies have obtained union by 3 months following surgery [3, 4, 11]. In this study, we compared classic radiographic follow up (immediate postoperative and at one, two, and 3 months) with computed tomographic follow up (immediate postoperative and at one, two, and 3 months) and ultrasonographic follow up assessment (at one, two, and 3 months). Ultrasonography was able to reliably diagnose complete bridging of the osteotomy gap, and provided a subjective diagnosis of complete bony bridging of the osteotomy gap at 1 month in eight out of 10 dogs, which was significantly earlier than for CT or radiographs. The ultrasonographic diagnosis of confluent bridging bone in the osteotomy gap at 1 month provides further evidence that healing may be further advanced than radiographs show, or rather that radiographic evidence of healing lags behind bone healing. This is similar to earlier reported studies investigating the use of ultrasonography in the follow up of fracture healing, where ultrasound reliably indicated bridging of the fracture gap with mineralized tissue at an earlier time point than radiographs [15, 16, 18, 21, 24–26]. One limitation is not obtaining a base line ultrasound image of the additives in the osteotomy gap immediately after surgery. The presence of air in the surgery site would have caused artifacts, limiting assessment of any tissues and structures deep to the air [31]. Ultrasonographic union was diagnosed on the appearance of a full bridge of mineralized tissue with acoustic shadowing deep to it, as defined during earlier studies assessing secondary fracture healing [15]. During secondary fracture healing, small areas of mineralization could be detected prior to formation of a full osseous bridge with return of acoustic shadowing [15]. Individual bone chips could have mimicked these foci of healing, but would not be visualized as a full osseous bridge.

Bone growth and regrowth are important clinical factors after orthopedic procedures involving osteotomies, ostectomies or after fracture stabilization. Using modalities that can diagnose healing reliably at an earlier stage could allow earlier rehabilitation and earlier return to full function postoperatively. Diagnosing or recognizing a delay in bone ingrowth or bone bridging will allow an earlier intervention to address the etiological cause for the delay in healing. Delayed healing of osteotomy sites will delay the return to normal function of the animal and healing complications after fracture treatment might be potentially devastating.

The CT studies provided more information than the radiographic studies, and could be performed during the same sedation with no need for general anesthesia. CT diagnosed complete healing by 2 months in eight out of the 10 dogs, which was not significantly earlier than the radiographic diagnosis. The current study found a significant increase in HUs over time, which is similar to a recently reported prospective study of antebrachial fractures [18]. However, using this objective method (quantifying the extent of mineralization in the developing osseous tissues by measuring HUs) did not provide a reliable method for diagnosing or predicting healing in this limited case series. One potential reason might be the continued presence of cortical chips in the DBM mix group in the earlier postoperative time, falsely increasing the HUs measurement. Another possible explanation might be the fact that for an accurate measurement only the tissues within the osteotomy gap should be included without capturing any adjacent soft tissues or bone, leaving only a very small area to be assessed. While using CT might not be indicated for routine follow up, it might provide additional information to the classic radiographic evaluations in selected cases.

Similarly, using the Hoffman radiographic scoring system did not add additional predictive value to the subjective healing assessment [4]. Only one dog in the current study had obtained a complete radiographic union by the end of the study (i.e. a score of ‘4’; bone bridging at all three sites). This is similar to the findings in the original paper by Hoffman et al. where five out 52 patients (10%) were given a score of ‘4’ (at a mean of 11.4 weeks postoperatively) [4]. The scores were not significantly different from the subjective healing assessment (*p* = 0.052) if we used bridging at two out of three sites as the definition of osseous union.

The Radiographic Union Score for Tibial fractures (RUST) described by Whelan et al. was also considered as a possible objective measurement to evaluate radiographic bone healing [32]. The RUST score was devised and tested specifically in tibial fractures treated with intramedullary fixation, allowing assessment of all four cortices. Using orthogonal radiographs, all four cortices are scored on a ‘1–3’ system [fracture line, no callus (score = ‘1’), fracture line, visible callus (score = ‘2’); no fracture line, bridging callus (score = ‘3’)] allowing for a total score ranging from ‘4’ to ‘12’. While this system was proven to have substantial intra observer agreement, it relies heavily on the visibility of bridging callus and cortical margins across four cortices and given the anatomy of the tibial tuberosity, the wedge shape of the osteotomy gap, and gap healing at this site, the authors felt that the RUST scoring system was not an applicable tool for evaluating effective bone formation in this study.

The bridging bone which formed in the osteotomy gap was consistently thinner than the width of the original osteotomy. However, the width of the bone in the GM group was significantly thinner (as thin as 2.5 mm in one dog) than the bone in the DBM mix group. When we created an objective parameter (ratio of bridge to adjacent bone) for comparison of the bridge width this finding proved non-significant (*p* = 0.10), most likely due to small sample size (power 36%, with a sample size of eight needed, to obtain a significant difference). In addition, the clinical relevance of the smaller bone bridge is unknown.

In contrast to an earlier study reported by Barnes et al., that used an aluminum stepwedge converted to mm aluminum equivalent, we did not find a significant difference for the tissue in the osteotomy gap during the follow up recheck radiographs. We used a direct comparison of the tissue in the fracture gap with the closest corresponding step of the wedge, while Barnes et al. used a non-linear regression analysis [11]. It therefore possible that our direct method of comparison was less sensitive than the model used by Barnes et al. [11].

The current study allowed for a direct comparison between two bone graft substitutes in an osteotomy gap model. The original TTA procedural description recommended placing an autogenous cancellous bone graft in the osteotomy gap; however, more recent studies have shown that a TTA osteotomy gap will progress to radiographic union without grafting [8, 10, 11]. We did not include a negative control group in which nothing was placed in the osteotomy gap, nor did we have a positive control group receiving an autogenous cancellous bone graft. Demineralized bone matrix had been and was routinely used when performing TTAs at our institution at the time that we initiated this study. We chose to compare the gelatinous matrix to the standard of care (DBM mix). The current study used the TTA osteotomy gap, in dogs with naturally occurring cranial cruciate ligament insufficiency that were otherwise healthy, to compare to commercially available bone graft substitutes’ potential for enhancing bone healing. While we did not find any significant differences between DBM mix and GM, most likely due to the small sample size, we did not observe any adverse effects in either treatment group. Due to its liquid form, GM may have a potential use in filling in smaller gaps, potentially through a key hole incision, in cases suspected to have, or at risk for, delayed bone healing.

## Conclusions

Our results confirmed that ultrasonography reliably predicted osseous union earlier than the other imaging modalities evaluated and CT provided additional information in comparison to radiographs. Dogs in both treatment groups uniformly obtained complete union within the 3 month time frame, although the clinical implications of the bone bridging the osteotomy gap in the GM treatment being thinner than that in the DBM mix group warrants further evaluation.

## Abbreviations

BW: Body weight
CR: Computed radiography
CT: Computed tomography
DBM mix: Demineralized bone matrix mix
GM: Gelatinous matrix
HM: Caudal hemimeniscectomy
HUs: Hounsfield units
MM: Medial meniscal treatment
MR: Meniscal release
NSAID: Non steroidal anti-inflammatory drug
PACS: Picture archive and communication system
PO: Postoperative
ROI: Region of interest
RUST: Radiographic union score for tibial fractures
TTA: Tibial tuberosity advancement
US: Ultrasonography
